# A cost-benefit/cost-effectiveness analysis of an unsanctioned supervised smoking facility in the Downtown Eastside of Vancouver, Canada

**DOI:** 10.1186/1477-7517-11-30

**Published:** 2014-11-13

**Authors:** Ehsan Jozaghi, Vancouver Area Network of Drug Users

**Affiliations:** School of Criminology, Simon Fraser University, 8888 University Drive, Burnaby, British Columbia V5A 1S6 Canada; 380 East Hastings Street, Vancouver, British Columbia V6A 1P4 Canada

**Keywords:** Supervised smoking facility, Crack, VANDU, Hepatitis C, Downtown Eastside

## Abstract

**Background:**

Smoking crack involves the risk of transmitting diseases such as HIV and hepatitis C (HCV). The current study determines whether the formerly unsanctioned supervised smoking facility (SSF)—operated by the grassroot organization, Vancouver Area Network of Drug Users (VANDU) for the last few years—costs less than the costs incurred for health-care services as a direct consequence of not having such a program in Vancouver, Canada.

**Methods:**

The data pertaining to the attendance at the SSF was gathered in 2012–2013 by VANDU. By relying on this data, a mathematical model was employed to estimate the number of HCV infections prevented by the former facility in Vancouver’s Downtown Eastside (DTES).

**Results:**

The DTES SSF’s benefit-cost ratio was conservatively estimated at 12.1:1 due to its low operating cost. The study used 70% and 90% initial pipe-sharing rates for sensitivity analysis. At 80% sharing rate, the marginal HCV cases prevented were determined to be 55 cases. Moreover, at 80% sharing rate, the marginal cost-effectiveness ratio ranges from $1,705 to $97,203. The results from both the baseline and sensitivity analysis demonstrated that the establishment of the SSF by VANDU on average had annually saved CAD$1.8 million dollars in taxpayer’s money.

**Conclusions:**

Funding SSFs in Vancouver is an efficient and effective use of financial resources in the public health domain; therefore, Vancouver Coastal Health should actively participate in their establishment in order to reduce HCV and other blood-borne infections such as HIV within the non-injecting drug users.

## Background

Smoking crack cocaine is not only on the rise in the Canadian municipalities, but it is also often neglected by health officials—especially so when compared to similar inner-city health problems such as injection drug use [1–4]. In British Columbia, the daily usage of crack cocaine within the general population is higher than that in any other provinces within Canada [5]. This is a pressing problem in Vancouver, where daily crack use, within a cohort of injection drug users, increased from 7.4% in 1996 to 42.6% in 2005 [6]. Among drug users in Vancouver’s Downtown Eastside (DTES), the rate of crack use has been reported to be as high as 86.6% [7]. The use of crack is associated with several other risks when compared to the tendencies displayed by other drug-using populations. For example, crack users are more likely to have unstable housing [8], be involved in sex work [9], participate in risky behavior [10–12], engage in criminal activity [13, 14], experience multiple health problems [5], and are less likely to access social and health services [15].

Research conducted upon a cohort of crack-user population in Vancouver’s DTES revealed that participants had reported 80% sharing rate as it is related to their drug smoking paraphernalia [16, 17]. Studies have shown a higher-than-average prevalence of human immunodeficiency virus (HIV), hepatitis C virus (HCV), and tuberculosis in users of crack cocaine who report no injection drug use [17]. However, the evidence of the relationship between non-injecting drug use and HIV/HCV infection is ambiguous. Some researchers have suggested that non-injecting drug users (NIDUs) are often involved in unsafe sexual behavior [18] and that HCV transmission in NIDUs is associated with tattooing [19]. Some researchers have stirred up a controversy in suggesting that NIDUs are essentially injecting drug users (IDUs) who have failed to report their route of transmission accurately [20].

Nevertheless, research conducted on NIDUs suggests that infectious diseases may have been transmitted by the sharing of crack pipes [21, 22]. In fact, most users are oblivious to the risks involved in sharing drug tools [21]. Some researchers postulate that HIV and HCV transmission can be accounted for by the high prevalence of oral lesions in crack smokers. Some of these include sores, blisters, and cuts on their lips and oral cavities—caused because of the mouth and lips coming in contact with hot glass, hot smoke, and the sharp edges of glass pipe stems or metal pipe stems [21]. The lack of knowledge with respect to transmittable diseases further engenders and reinforces the reckless exchange of drug equipment. In fact, a study demonstrated that 2% of crack pipes tested positive for HCV [23].

Scientific evaluation of Insite, North America’s first and only supervised injection facility, showed that it has successfully reduced needle sharing and overdose death while concurrently improving service uptake and public order within the DTES [24–26]. Despite the improvement of conditions in the DTES after the opening of Insite, Vancouver is still riddled with concerns regarding public health and order related to drug use, including crack and crystal methamphetamine [27, 28]. Accordingly, the region’s health authority has shown some interest in applying for an exemption under the *Controlled and Substance Act* of the *Criminal Code of Canada* to open a supervised smoking facility (SSF) in the DTES. However, the concept of a government-sanctioned SSF is somewhat controversial, particularly because the potential impact and benefits of such a facility are unknown.

**Figure 1:**
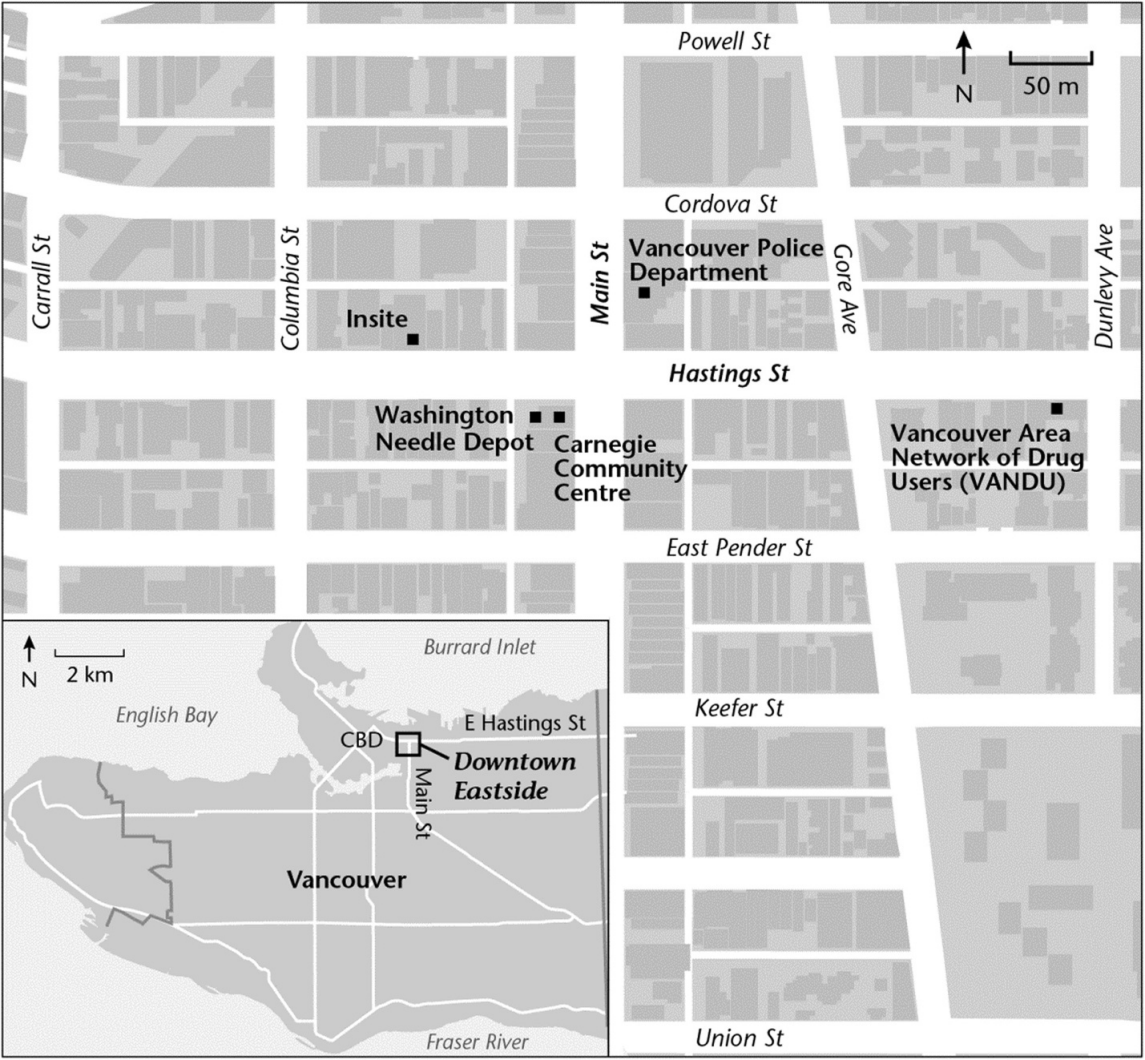
**Map of the DTES.**

Therefore, the present research was conducted to determine whether a case could be made for the establishment of SSFs in the DTES of Vancouver. Specifically, the current study analyzed the cost-benefits and cost-effectiveness of the only SSF in Canada, operated by Vancouver Area Network of Drug Users (VANDU) without a license for a few years. The SSF mentioned above was located in VANDU’s front office in the DTES, along East Hastings Street. VANDU has over 800 volunteers, 1,300 active members [29], and a Board of Directors composed of current and former users. See Figure 1 for the location of VANDU in the DTES.

In December of 2013, VANDU was forced to shut down the SSF under the direction of their funding agency, the Vancouver Coastal Health. Using mathematical modelling with conservative parameter estimates, this analysis estimated the number of HCV infections prevented as a result of SSF. The savings from illnesses avoided were compared to the operational cost of a SSF. The analysis was eventually extended to consider the impact of opening additional SSFs in the DTES.

## Methods

### Background

**Figure 2:**
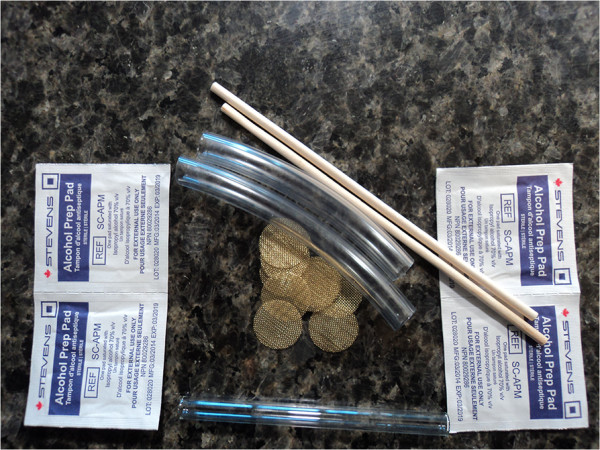
**Content materials of a ‘safer crack use kit’ provided at VANDU.**

VANDU operates on an annual budget of CAD$200,000 funded through Vancouver Coastal Health. One of their various programs included the operation of an unsanctioned SSF. The smoking room was operated by peers and was accessible to one person at a time. There would be an unusually big lineup to use the room that contained a fan. Within VANDU, NIDUs would generally be provided with a ‘safer crack use kit’ that contained the following: mouth pieces, wooden push sticks, screens, alcohol swaps, and heat-resistant and shatter-proof glass pipes which minimized chances of injury to the users’ lips and mouth. See Figure 2 for materials contained in the ‘safer crack use kit’ provided at VANDU.

Moreover, VANDU’s SSF provided a clean and safe environment within which one could use pre-obtained illicit drugs, get medical attention in the event of an overdose, and obtain access or referral to primary health care when required. This study was approved by Simon Fraser University Research Ethics Board (study number: 2013 s0058). VANDU’s Executive Board also approved the study since it corresponded with its philosophy and the demand that all projects directly involve its members.

### Model

For this analysis, it was necessary to calculate the effects of both providing clean equipment as well as that of adopting safer smoking behaviors. Along the lines of research conducted on the economic impact of a needle exchange program in Edmonton, Alberta, Canada, this study uses a mathematical model to estimate the number of HCV infections that could be prevented through the establishment of a SSF [30]. The number of new HIV infections avoided, *A*, is calculated as follows:


where *m* is the number of sharing partners when pipes are shared, *t* is the probability of HCV transmission when using an HCV-infected pipe, *s* is the rate of pipe sharing, *I* is the proportion of NIDU population that is HCV negative, *N* is the number of pipes in circulation, *d* is the percentage of pipes not cleaned before use, and *q* is the proportion of the NIDU population that is HCV positive.

Initially, this study was meant to use a few other mathematical models such as those of Kaplan and O’Keefe [31], Lurie and Drucker [32], Gold et al. [33], Laufer [34], and Pinkerton [35, 36]. However, due to lack of data, such as the rate of HIV transmission from a single pipe and the rate of secondary transmission, this analysis had to rely on the Jacobs et al. [30] model. However, the model employed in the current study has previously been adopted by four different studies [37–40], which have found that this model is the best choice for predicting actual and potential cases of HIV and HCV in a Canadian setting. Moreover, the model employed in this study has successfully produced estimates of HIV and HCV cases within the IDU population—similar to known data widely cited in peer-reviewed reports.

Additionally, this study also uses behavior change incorporated by previous costing studies conducted on supervised injection facilities [37–41] because of the empirical evidence it provides [24, 35, 41, 42]. Although previous costing studies often go wrong when it comes to using caution and employing an odd ratio of 0.60, this study uses the point estimate of 0.30 used by previous studies [37–42] and estimated by Kerr et al. [24]. The data collected by VANDU during 2012–2013 pertains to the number of visits per month to the SSF.

### Variables and parameters

Medical and scientific literatures were used in cases when Vancouver-specific data was not available. Where estimates differed, this study used the lower bound, so all estimates remain conservative. The concept of behavioral change in the NIDU population was adopted based on the behavioral changes related to IDU needle-sharing behavior outside of the SIF in Vancouver. Kerr et al. [24] and Bravo et al. [43] found that IDUs who relied upon SIFs were also able to reduce their needle-sharing activities outside of the facility up to a significant extent. Table 1 provides the estimates and variables used in the model (please note that percentages need to be converted to fractions when imputing the variables in the model).

**Table 1:** **Sources for variables used in mathematical modelling**

Variable	Value	Source
Rate of pipe sharing (*s*)	80.0%	Ivsins et al. [44]
Number of pipes in circulation (*N*)	90,000	VANDU [45], Mui [46]
Percentage of pipes not cleaned (*d*)	33.0%	Scheinmann et al. [47]
Number of sharing partners (*m*)	6.30	Gyarmathy and Neaigus [48]
Proportion of crack users who are HCV negative (*I*)	83.0%	Fischer et al. [49]
Proportion of crack users who are HCV positive (*q*)	17.0%	Fischer et al. [49]
Probability of HCV infection from single crack pipe (*t*)	2.00%	Fischer et al. [23], Gilbert et al. [50]

Consequently, it was presumed that NIDUs that visited the unsanctioned SSF were less likely to share their pipes with others outside of the facility. Furthermore, if a second SFF was established, the behavioral change on pipe sharing would occur only if new NIDUs became users of SSF [28]. On the contrary, if the SSF was frequented by the current users, thereby restricting its use to current users simply indulging in some additional smoking, no further behavioral changes can be assumed. Accordingly, behavioral change is only accounted for in the second facility.

Furthermore, since there was no estimated number of crack users in the DTES of Vancouver, this number was calculated based on the percentage of drug users (conservatively estimated to be around 5,000 in the DTES [51–53]) who have smoked crack. The total number of drug users was reported by DeBeck et al. [7] to be 86.6% (5,000 drug users × 0.866 use crack = 4,330 crack users in the DTES). This number was subsequently multiplied by the number of subjects who smoked crack per day—estimated to be around ten per day [54, 55] (4,330 × 10 × 365 days =15,804,500 smoking per year).

The number of those indulging in smoking per year was multiplied by the percentage of pipe sharing in the Downtown Eastside (15,804,500 smoking per year × 0.80 sharing = 12,643,600 shared crack smoking events). The total visits to the SSF during 2012–2013 year were determined to be 23,120 per year with the average visit of 1,843 per month. Consequently, 17,696 smoking incidents were not shared as a result of having an unsanctioned SSF operating in the DTES (23,120 × 0.8 sharing =17,696 smoking events that were not shared). This number was added to the behavioral change odd ratio and later deducted from the total shared crack pipe events in the DTES.

### The medical cost of new HCV cases

HCV infection among people who use drugs is a serious pressing concern in Canada and the United States [56, 57]. HCV infections could lead to multiple health problems such as cirrhosis, liver failure, hepatocellular carcinoma, and even death [58]. Accordingly, 50% of patients achieve sustained virological success to treatment [59]. Pegylated interferon, in combination with ribavirin, is the standard course of treatment for HCV-infected patients [57]. The range of treatment for HCV patients is determined based on the genotype: ‘a 48-week course is recommended for genotypes 1 and 4, whereas a 24-week course is recommended for genotypes 2 and 3’ [57], p. 1016. Accordingly, the cost of treatment varies according to genotype and seriousness of infections.

On an average, savings from HCV range from $20,000 per completed course of treatment per patient [60], to $30,000 [61], and to more than $69,188 [58]. This study uses a conservative figure of CAD$35,143 (2012 US Dollars = 33,856), as reported in [62] and utilized in costing studies of a potential SIF in Montreal [39] and Ottawa [40]. The conservative figure used in this study essentially disregards the cost of the complications arising from HCV in hepatocellular carcinoma, liver failure, and liver transplant cases.

### Cost of SSF

In order to estimate the cost of operating a potential SSF, it was important to calculate the operating cost of the existing SSF in the DTES. The former facility operated from Monday to Friday from 10–7 pm. On weekends, the facility would operate from 4–7 pm. The staff supervising the unsanctioned SSF were mostly volunteers that were provided with a small stipend, collectively amounting to CAD$47,203 per year. The total cost of the rent and the safe crack kit is estimated to be CAD$50,000. Altogether, the operating cost of the facility is estimated to be CAD$97,203.

## Results

The model used here [26] predicted the number of new HCV cases prevented based on the pipe-sharing rate. This included the impact of behavioral changes in pipe sharing outside of the SSF. The behavioral change, according to Tables 2 and 3, was only considered twice—once for the first SSF and once for the second SSF—based on a conservative odd ratio that falls within the limit specified by [31].

**Table 2:** **The cumulative cost-effectiveness and cost-benefit of SSF in Vancouver using Jacobs et al.’s** [30] **model**

Variables	Annual cost of operation ($)	Sharing rate (%)	# of HCV averted	Cost-effectiveness ratio HCV ($)	Cost-benefit ratio HCV
Post SSF	97,203	69	57	1,705	20.6
		(78, 60)	(65, 50)	(1,495, 1,944)	(23.5, 18.1)
Two SSF	194,406	59	109	1,784	19.7
		(67, 52)	(121, 93)	(1,607, 2,090)	(21.9, 16.8)
Three SSF	291,609	58	110	2,651	13.3
		(67, 52)	(121, 94)	(2,410, 3,102)	(14.6, 11.3)
Four SSF	388,812	58	111	3,503	10
		(67, 52)	(122, 94)	(3,187, 4,136)	(11, 8.5)
Five SSF	486,015	58	112	4,339	8.1
		(67, 52)	(123, 95)	(3,951, 5,116)	(8.9, 6.9)
Six SSF	583,218	57	113	5,161	6.8
		(66, 52)	(124, 95)	(4,703, 6,139)	(7.5, 5.7)
Seven SSF	680,421	57	114	5,969	5.9
		(66, 52)	(124, 96)	(5,487, 7,088)	(6.4, 5)

The numbers in parentheses represent the results of the sensitivity analysis (90% sharing rate, 70% sharing rate).

**Table 3:** **The marginal cost-effectiveness and cost-benefit of SSF in Vancouver using Jacobs et al.’s** [30] **model**

Variables	Annual cost of operation ($)	Sharing rate (%)	# of HCV averted	Cost-effectiveness ratio HCV ($)	Cost-benefit ratio HCV
Post SSF	97,203	69	57	1,705	20.6
		(78, 60)	(65, 50)	(1,495, 1,944)	(23.5, 18.1)
Two SSF	97,203	59	52	3,739	18.8
		(67, 52)	(56, 43)	(1,736, 2,261)	(20.2,15.5)
Three SSF	97,203	58	1	97,203	0.4
		(67, 52)	(1, 1)	(97,203, 97,203)	(0.4, 0.4)
Four SSF	97,203	58	1	97,203	0.4
		(67, 52)	(1, 0.5)	(97,203, 194,406)	(0.4, 0.2)
Five SSF	97,203	58	1	97,203	0.4
		(67, 52)	(1, 0.5)	(97,203, 194,406)	(0.4, 0.2)
Six SSF	97,203	57	1	97,203	0.4
		(66, 52)	(1, 0.5)	(97,203, 194,406)	(0.4, 0.2)
Seven SSF	97,203	57	1	97,203	0.4
		(66, 52)	(1, 0.5)	(97,203, 194,406)	(0.4, 0.2)

The numbers in parentheses represent the results of the sensitivity analysis (90% sharing rate, 70% sharing rate).

As expected, the results presented in Tables 2 and 3 show that expanding SSFs would decrease HCV cases. The model predicts 57–114 cases for HCV with the marginal range being much smaller at 1–57 for HCV.

This range disparity, as outlined in Tables 2 and 3, translates into substantial differences between the cumulative estimates and the marginal estimates. For example, according to Table 2, the benefit-cost ratio ranges from 5.9 to 20.6 and the cost-effectiveness value ranges from $1,705 to $5,969 (cost per lifetime treatment). In contrast, the marginal estimates of SSF expansion translate into a much smaller return. This is particularly true with respect to its benefit-cost and cost-effectiveness ratio; for instance, the marginal benefit-cost ratio varies from 20.6 to 0.4. The marginal cost-effectiveness value for HCV ranges from $1,705 to $97,203 (cost per lifetime treatment). Furthermore, Table 3 shows that both cumulative benefit-cost and cost-effectiveness ratios dwindle after the second SSF.

Finally, a sensitivity analysis was conducted for the models employed. The sensitivity analysis pertained to simulating different pipe-sharing rates (see Tables 2 and 3). Similar to costing studies in Vancouver [37, 38], Montreal [39], and Ottawa [40] that used different needle-sharing rates, the current analysis used 70% and 90% initial pipe-sharing rates. Convincingly, the results from both the baseline and sensitivity analysis demonstrate that the establishment of an SSF by VANDU had saved taxpayer money.

## Discussion

The current analyses assessed whether the former SSF, operated by VANDU in the DTES, would have had a net positive fiscal impact on the Canadian society and whether or not this policy initiative would save public health-care funds by averting new HCV infections. Moreover, the optimal number of SSFs was assessed based on marginal cost-effectiveness and benefit-to-cost ratios. The results presented here suggest that closing the only unsanctioned SSF in Vancouver was a policy failure that has potentially resulted in the spread of HCV within the drug-user population. In fact, establishing more SSFs in Vancouver’s DTES would be a beneficial and fiscally responsible in addition to the publically funded health-care system. Based on the marginal counts, it should be noted that although expansion beyond the second SSF location may not provide the same economic return as the cumulative estimates, it may still be considered cost-effective given that the cumulative result was cost-effective beyond the seventh potential location.

Though not outlined in this analysis, there are several other benefits of opening a SSF that may add to the existing financial benefits of a SSF. One such benefit is the lowering of the risk of overdose, particularly for those smoking heroin and methamphetamine [63]. In British Columbia alone, 14 deaths have been attributed to heroin smoking [27]. Given the medical supervision of NIDUs, SSF has the potential to mitigate the risk of overdose deaths.

Another benefit of opening a SSF is the potential to increase detoxification and reduce risk behavior through education. Research indicates that NIDUs will change their risk behavior when provided with appropriate education and treated with care [27]. Moreover, IDUs that regularly use the Vancouver’s Insite are more likely to initiate and maintain addiction treatment [4]. By visiting a SSF, people who use drugs may utilize various services such as mental health, counselling, and detoxification. Furthermore, SSFs can be expected to reduce public drug use in the same way that Vancouver’s Insite has been able to reduce public drug-use behavior of IDUs.

In summary, not only on the use of crack among drug users is on the rise but also the sharing of crack pipe has been increasing at an alarming rate in Vancouver. With recent research demonstrating the significant risk of disease transmission via oral smoking equipment, the current study determined whether the former unsanctioned SSF operated by the grassroot organization, VANDU, would cost less than the health-care consequences of not having such a program in Vancouver. The results indicated that the former facility not only saved taxpayers’ money but also deserved to be expanded instead of being forced to shut down. This information and analysis should be useful for policy makers who seek to find practical, cost-effective solutions to serious health-care problems in a climate of scarce public resources.

## Abbreviations

VANDU: Vancouver Area Network of Drug Users
DTES: Downtown Eastside
SSF: supervised smoking facility
HCV: hepatitis C
IDU: injecting drug user
NIDU: non-injecting drug user
HIV: human immunodeficiency virus.
